# Exacerbation of Eosinophilic Granulomatosis With Polyangiitis After Administering Dupilumab for Severe Asthma and Eosinophilic Rhinosinusitis With Nasal Polyposis

**DOI:** 10.7759/cureus.25218

**Published:** 2022-05-22

**Authors:** Shunya Tanaka, Taisuke Tsuji, Shinsuke Shiotsu, Tatsuya Yuba, Noriya Hiraoka

**Affiliations:** 1 Respiratory Medicine, Japanese Red Cross Society Kyoto Daiichi Hospital, Kyoto, JPN

**Keywords:** bronchial asthma, chronic rhinosinusitis with nasal polyposis, hyper-eosinophilia, dupilumab, egpa

## Abstract

Eosinophilic granulomatosis with polyangiitis (EGPA) refers to systemic vasculitis in patients with bronchial asthma and eosinophilic rhinosinusitis. Dupilumab has been approved for the treatment of asthma, eosinophilic rhinosinusitis, and atopic dermatitis. A man in his 50s with a history of asthma and eosinophilic rhinosinusitis with nasal polyposis developed high fever and dyspnea while undergoing dupilumab treatment. Laboratory examinations identified hypereosinophilia. Chest radiography and computed tomography revealed right-sided tracheal dislocation, extensive consolidation, and ground-glass opacities with traction bronchiectasis. Evidence of interstitial pneumonia, eosinophilia, and increased eosinophil counts in the bronchoalveolar lavage fluid was observed. We diagnosed the patient with EGPA and administered corticosteroids, which improved his symptoms and radiographic signs. Although the relationship between EGPA and dupilumab treatment is unclear, EGPA may have been exacerbated by dupilumab in this case. Therefore, when administering dupilumab to patients with partial symptoms of EGPA, care should be taken to monitor for adverse symptom development and exacerbation.

## Introduction

Dupilumab, which was approved by the Food and Drug Administration in 2017, is a fully human, monoclonal, immunoglobulin (Ig) G4 subclass antibody that blocks the interleukin-4 receptor alpha (IL-4Rα) subunit of the interleukin (IL)-4 and IL-13 receptors. By binding IL-4Rα, dupilumab inhibits IL-4 and IL-13 signaling pathways, which are the principal drivers of type 2 inflammation in conditions such as chronic rhinitis, bronchial asthma, and atopic dermatitis. The clinical efficacy of dupilumab for the treatment of asthma and chronic sinusitis has been confirmed [1,2].

Eosinophilic granulomatosis with polyangiitis (EGPA) is a multisystem disorder characterized by allergic rhinitis, asthma, and prominent peripheral blood eosinophilia. EGPA is classified as a vasculitis of small- and medium-sized arteries, although vasculitis is often not apparent in the initial phases of the disease. In the active phase of EGPA, T-helper cell type 2 cytokines such as IL-4 and IL-5, but also suppressive cytokines such as IL-10, are increased. IL-5 regulates innate immunity and natural inflammation, and its inhibition in the active phase of EGPA could represent a potential therapeutic target [3].

In our practice, we have encountered patients who developed EGPA despite the inhibition of IL-4 and IL-13 during dupilumab treatment, and dupilumab may have played a role in the development of this condition. Herein, we discuss the cytokines involved and report one such case.

## Case presentation

A man in his 50s, with no history of smoking, was admitted to our hospital with an approximately two-week-long history of high-grade fever and dyspnea. For asthma and eosinophilic rhinosinusitis, he was treated with a leukotriene receptor antagonist, antihistamine, and inhaled glucocorticoid plus a long-acting β2-agonist. He did not take oral corticosteroids. Approximately five months before the emergency room visit, he was additionally started on dupilumab every two weeks for eosinophilic rhinosinusitis with nasal polyposis and intractable asthma.

His other medications had not changed during the six months leading up to the presentation. He had slight tachypnea, with oxygen saturation (SpO2) of 92% without oxygen supplementation. His temperature was 38.5°C, pulse was 102 bpm, and blood pressure was 117/81 mmHg.

**Figure 1:**
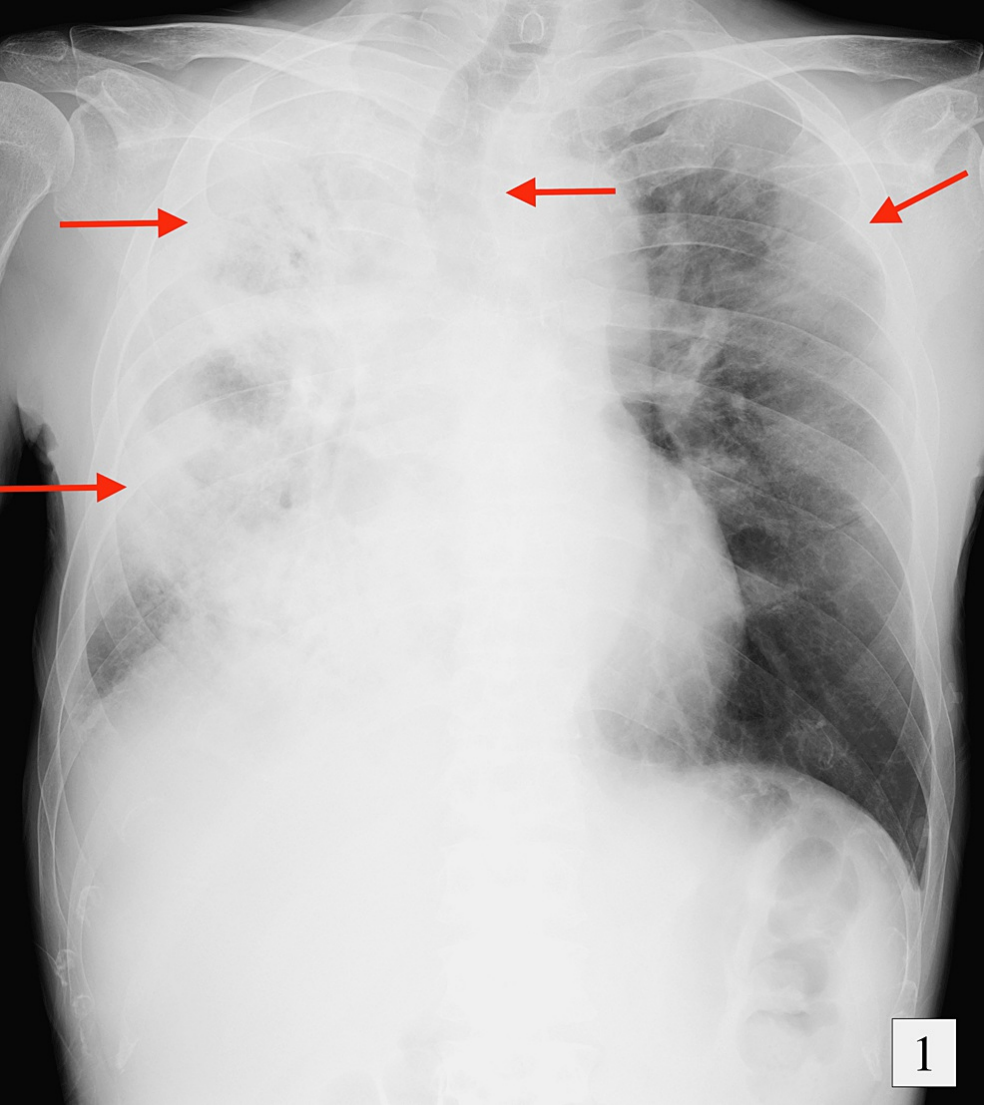
Chest radiograph on the first day of hospitalization showing tracheal excursion to the right side and consolidation in the right lung.

**Figure 2:**
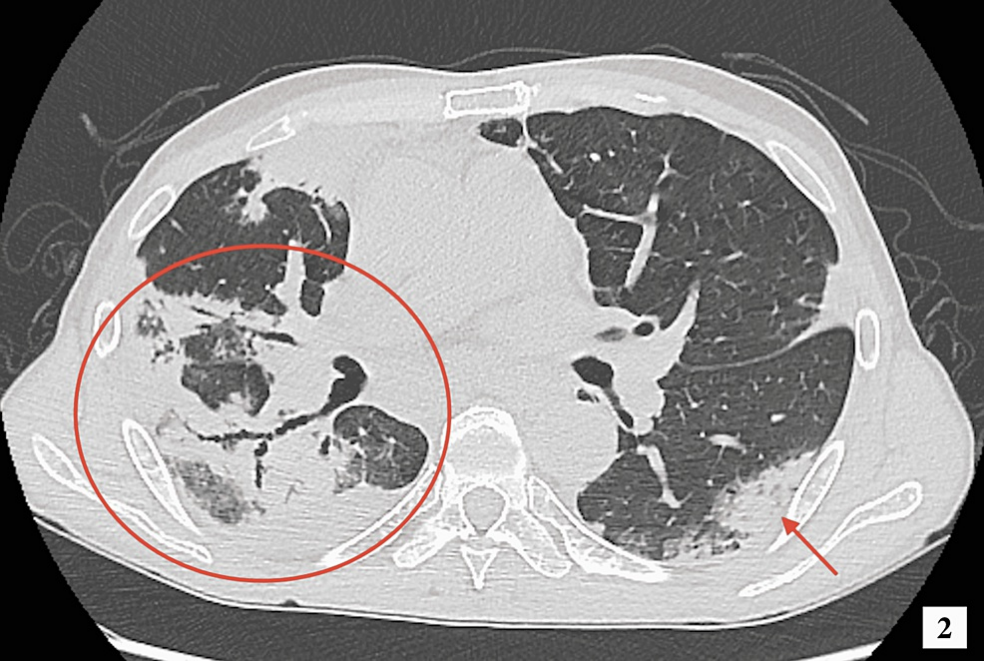
Computed tomography scan of the thorax showing extensive consolidation and ground-glass opacities with traction bronchiectasis.

On physical examination, lung auscultation revealed wheezing breath sounds in all lung zones. Further physical examination did not reveal any additional abnormalities such as rashes, muscle pain, or neuropathy. Laboratory testing returned a white blood cell count of 28.5 x 109/L, a neutrophil count of 8.3 x 109/L, and an eosinophil count of 17.4 x 109/L. There was no abnormality in liver and renal functions. The C-reactive protein (CRP), sialylated carbohydrate antigen KL-6, immunoglobulin E (IgE), and IL-5 were elevated to 163.2 mg/L, 301 U/mL, 930 mg/L, and 14.9 pg/mL, respectively. β-D-glucan and anti-parasitic antibody tests were negative. Immunological tests detected no positive antibodies, including myeloperoxidase-antineutrophil cytoplasmic antibodies (MPO-ANCA). Blood and sputum cultures were negative for bacteria and fungi. Chest radiography indicated that the trachea was displaced to the right and showed patchy opacity, and there was consolidation in the right lung. Chest computed tomography (CT) revealed extensive consolidation and ground-glass opacities with traction bronchiectasis (Figures 1, 2). Echocardiography showed centripetal left ventricular hypertrophy. There were no asynergy and valvular diseases.

**Figure 3:**
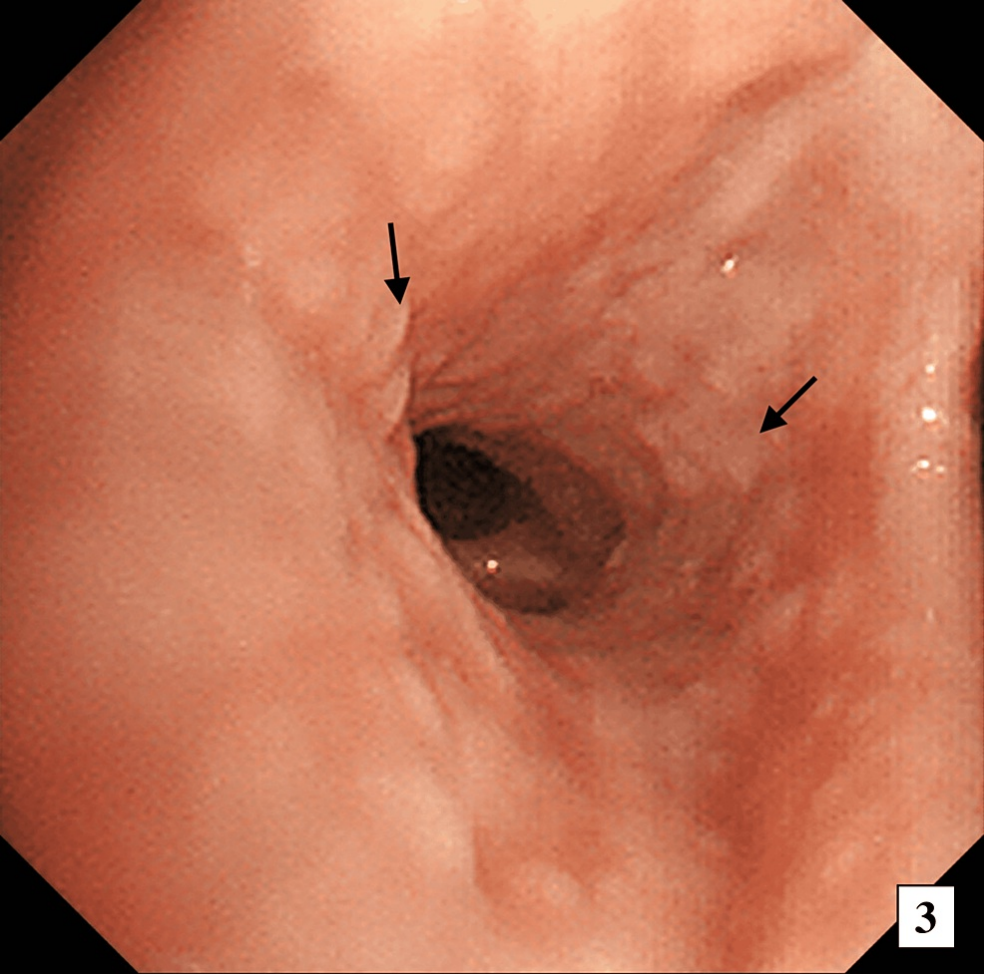
Flexible bronchoscopy showing multiple nodular lesions of the trachea and bronchi.

On the second day of hospitalization, bronchoalveolar lavage (BAL) and transbronchial lung biopsy revealed multiple nodular lesions of the trachea and bronchi (Figure 3). In the BAL fluid, the proportion of eosinophils was increased to 38%, and the proportion of neutrophils and lymphocytes was 6% and 1%, respectively. CD4/CD8 ratio was 1.79. BAL cultures and molecular diagnostic tests indicated no bacteria, fungi, or malignancy. A lung pathology specimen showed interstitial pneumonia with eosinophilia and no vasculitis. To exclude other causes of eosinophilia, a bone marrow biopsy and genetic testing were conducted. The bone marrow biopsy specimen revealed eosinophils occupying >50% of the bone marrow, but no abnormalities in differentiation. Genetic tests for BCR-ABL1, FIP1-like-1-platelet-derived growth factor receptor alpha (FIP1L1-PDGFRA), and fibroblast growth factor receptor 1 (FGFR1) were negative. Therefore, based on the 1990 American College of Rheumatology criteria, we diagnosed the patient with EGPA.

After bronchoscopy, his respiratory condition deteriorated, and he was provided respiratory support using a high-flow nasal cannula. High-dose methylprednisolone (1000 mg) was immediately administered and then continued at the same dose (1000 mg/day) for three days. His respiratory condition improved significantly after high-dose methylprednisolone therapy, and he was transitioned to oral prednisolone (PSL) at 60 mg/day (1 mg/kg) as a maintenance dose.

**Figure 4:**
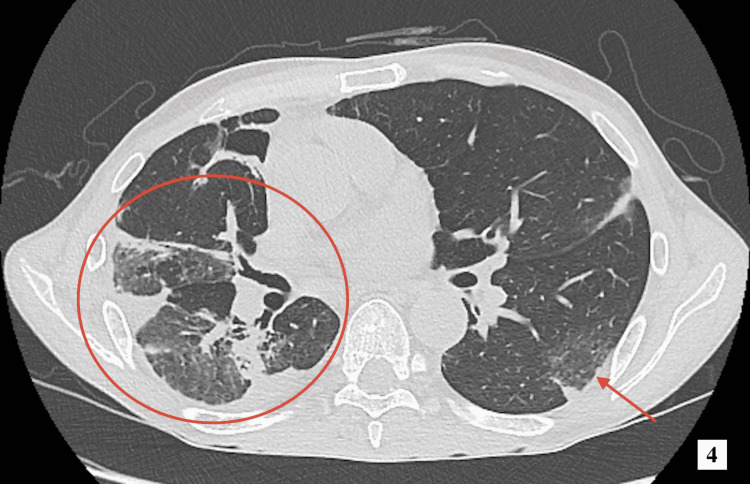
Computed tomography scan of the thorax showing improvement of bilateral opacification and traction bronchiectasis.

On the fourth day of hospitalization, his eosinophil level normalized and his CRP level dropped to 7.97 mg/dL. Follow-up chest CT on hospitalization day 10 showed a decrease in the appearance of bilateral opacification and traction bronchiectasis (Figure 4). His response to corticosteroid monotherapy was significant. Before corticosteroid administration, the patient’s Birmingham Vasculitis Activity Score was 12 points, which improved to 4 points post-treatment. On the 11th day of hospitalization, the PSL dose was reduced to 50 mg/day. His general condition improved significantly, and he was discharged on day 19.

**Figure 5:**
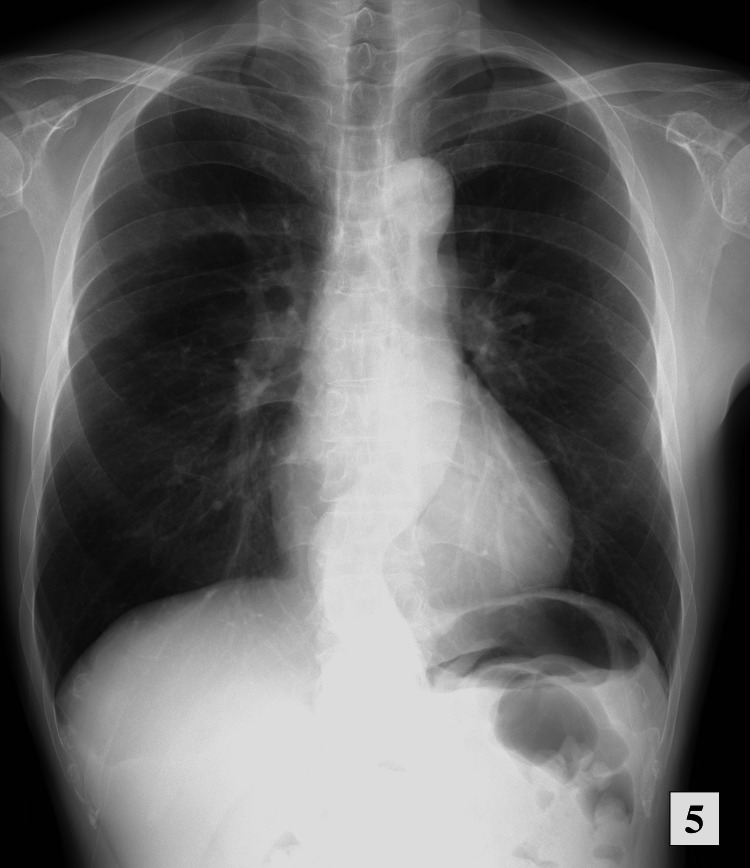
Chest radiograph five months after discharge showing significant improvement and no recurrence.

Currently, the PSL dose is tapered to 3 mg, and the patient attends regular follow-ups. The most recent chest radiograph showed significant improvement in tracheal dislocation and gradual disappearance of the consolidation and ground-glass opacities (Figure 5). There has been no recurrence of EGPA.

## Discussion

Eosinophilia is typically triggered by allergens, drugs, infection or inflammation, asthma, and tumors. In this case, there was no history of contact allergens, no history of changing or introducing new drugs aside from dupilumab, and no evidence of malignant disease or infection. In the present case, the patient’s eosinophil levels increased in the five months after starting dupilumab treatment. Similarly, in previous reports, eosinophilia occurred four to six months after dupilumab treatment initiation [4,5].

Eosinophils migrate out of circulation and into tissues via stepwise interactions with endothelial cells. The rolling of circulating eosinophils on the endothelium is primarily mediated by P-selectin. Subsequently, eosinophils adhere firmly to the endothelium via adhesion molecules of the integrin family: the CD18 family (β2 integrins) and very-late-antigen-4 (VLA-4) molecules (β1 integrins). β2 integrin interacts with intercellular adhesion molecule 1 (ICAM-1) on endothelial cells, while β1 integrin interacts with vascular cell adhesion molecule 1 (VCAM-1). The β2-ICAM-1 pathway is used by all types of white blood cells, but the VLA-4-VCAM-1 pathway is specific for eosinophils and monocytes and is activated by IL-4 [6]. Dupilumab blocks IL-4 signaling and inhibits the VLA-4-VCAM-1 pathway. When this pathway is inhibited, eosinophils cannot migrate into surrounding tissues, and they accumulate in the circulating blood. In the previous report, the eosinophil count was under 5.0 x 109/L in eosinophilia after administering dupilumab [5]. In our case, the eosinophilic count was 17.4 x 109/L. Based on our patient’s timeline for eosinophilia onset, it is possible that the condition was induced by dupilumab treatment in the present case.

Dupilumab indirectly affects the production of IL-5 by inhibiting the binding of IL-4 and IL-4Rα. T-helper lymphocytes of Th2 and group-2 innate lymphoid cells (ILC2) produce IL-5. IL-4 promotes cytokine production via ILC2 through direct interaction with IL-4Rα [7]. Therefore, after starting dupilumab treatment, the activity of ILC2 should be suppressed, and the IL-5 level should decline. Nevertheless, in this case, the IL-5 level was mildly elevated. IL-5 is typically elevated in diseases that cause strong eosinophilic inflammation, including eosinophilia, EGPA, and eosinophilic pneumonia. We hypothesized that the effects these diseases had on IL-5 elevation exceeded the suppressive effect of dupilumab.

In this case, the diagnosis was considered controversial based on the classification of antineutrophil cytoplasmic antibody (ANCA)-associated vasculitis and polyarteritis nodosa developed by the 1990 American College of Rheumatology [8]. To diagnose EGPA, four or more of the following six criteria need to be met: (1) asthma, (2) eosinophilia of >10% on differential white blood cell count, (3) mononeuropathy (including multiplex) or polyneuropathy, (4) non-fixed pulmonary infiltrates on roentgenography, (5) paranasal sinus abnormality, and (6) extravascular eosinophils on histology. Our patient met four of these criteria and was therefore diagnosed with EGPA. The differential diagnoses, including eosinophilic pneumonia or non-classified eosinophilic disorder, were eliminated based on a collective consideration of the clinical, serological, and genetic data, and imaging tests.

Previous reports described some patients who had onsets of EGPA after being administered dupilumab [2,9]. In these reports, some cases became severe. It is unclear how dupilumab induces EGPA. We considered that EGPA was latent and became symptomatic after the administration of dupilumab by chance. The clinical course of EGPA is stratified into three phases. In the first phase, patients frequently develop asthma and rhinosinusitis, and sometimes atopy. In the second half of the first phase, blood eosinophilia appears, and MPO-ANCA tests sometimes become positive. In the secondary phase, systemic vasculitis occurs rapidly, and the clinical features include vasculitis processes such as neuropathy, purpura, scleritis, and glomerulonephritis. The last phase is the long-term follow-up period [10]. In the current case, dupilumab was administered in the first half of the first phase, which may have caused the latent EGPA to surface.

In this case, the patient was aware of the symptoms for several weeks leading up to the presentation. If he had undergone laboratory testing or chest radiography earlier, he may have received treatment before the exacerbation of symptoms began. Therefore, it is necessary to confirm whether a patient has a physical appearance or serological and radiological features of vasculitis and eosinophilia before and after administering dupilumab for asthma, rhinosinusitis, and atopy. Performing chest radiography and blood tests (MPO-ANCA, proteinase 3 (PR3)-ANCA, and blood eosinophils) in advance could be useful for ruling out these diseases during differential diagnoses. After dupilumab administration, it may be necessary to regularly monitor the blood eosinophil count and perform chest radiography. When the blood eosinophil count increases or symptoms of vasculitis appear, dupilumab treatment should be stopped.

This report has some limitations. First, we could not confirm EGPA pathologically. Instead, the diagnosis of EGPA was based on the 1990 American College of Rheumatology criteria of asthma, eosinophilia > 10% of the differential white blood cell count, non‐fixed pulmonary infiltrates on roentgenography, and paranasal sinus abnormality. Therefore, the diagnosis was controversial. Second, we could not measure other cytokine levels, such as IL-4 and IL-13, which are involved in eosinophilia. These cytokines may have played an important role in this case and may have provided some further supporting pathological information. Third, it is unclear about the transition of fractional exhaled nitric oxide (FeNO), IL-5, eosinophils count, and questionnaires on asthma and rhinosinusitis such as the Asthma Control Test (ACT), Asthma Control Questionnaire-5 (ACQ-5), and Sino-Nasal Outcome Test-22 (SNOT-22) before and after the administration of dupilumab.

## Conclusions

We experienced a case of EGPA after dupilumab administration. After dupilumab administration, blood eosinophil levels sometimes increase. However, it is unclear whether the eosinophilia is directly induced by dupilumab or caused by the activation of latent diseases like EGPA. Eosinophilia can be critical when it causes acute respiratory failure and myocarditis. Therefore, it is necessary to monitor a patient’s eosinophil levels regularly after administering dupilumab. It is especially necessary to closely monitor patients with partial symptoms of EGPA (e.g., asthma and sinusitis) during dupilumab treatment and ensure careful follow-up with physical examination, blood tests, and radiography.
